# Real-Time Physical Activity Self-Monitoring in Patients Hospitalized for Cardiology Care: Interrupted Time Series Analysis

**DOI:** 10.2196/92189

**Published:** 2026-10-07

**Authors:** Carlos de Miguel Llorente, Gertjan T J Sieswerda, Kaoutar Karramass, Charlotte Rommens, Thomas J Hoogeboom, Karin Valkenet

**Affiliations:** 1 Department of Rehabilitation, Physiotherapy Science and Sport, University Medical Center Utrecht, Heidelberglaan 100, Utrecht, 3584 CX, The Netherlands; 2 Department of Cardiology, University Medical Center Utrecht, Utrecht, The Netherlands; 3 Group Innovation of Human Movement Care, University of Applied Sciences Utrecht, Utrecht, The Netherlands

**Keywords:** inpatients, motor activity, self-monitoring, implementation science, cardiology

## Abstract

**Background:**

Hospital admission is associated with increased sedentary behavior and low levels of physical activity. Hospitals have developed several strategies and interventions to address this unwanted inactivity and increase patient movement during admission. Self-monitoring of physical activity is a promising approach to support activity during hospital stays.

**Objective:**

This study investigated whether providing patients with real-time physical activity feedback, compared to a context where only health care practitioners had access to activity data, supported patients in maintaining activity levels in the cardiology ward.

**Methods:**

A Hybrid Type 2 interrupted time series design was applied. In phase 1 (24 wk), patients wore accelerometers (Physical Activity Monitor [PAM] AM400) with data visible only to health care professionals. In phase 2 (24 wk), self-monitoring was introduced using a ward-based screen that provided patients with real-time feedback on daily physical activity. Implementation outcomes were evaluated within the RE-AIM (Reach, Effectiveness, Adoption, and Implementation) framework, with “Maintenance,” defined as daily physical activity trends over time, serving as the primary outcome. The other RE-AIM dimensions were assessed as secondary outcomes.

**Results:**

A total of 159 patients were included (n=75 in phase 1 and n=84 in phase 2). Daily physical activity levels were expressed as active minutes per day. No significant immediate change in daily activity occurred at the start of phase 2 vs the end of phase 1 (β=–0.127, *P*=.81). In phase 1, physical activity declined statistically significantly over time (β=–0.002, *P*<.001; ~6% decrease per month). In phase 2, following the introduction of the self-monitoring intervention, this decline was no longer observed, and activity levels were maintained. A significant phase interaction (β=0.002, *P*=.03) confirmed stabilization of physical activity levels in phase 2. Secondary RE-AIM outcomes did not differ between phases.

**Conclusions:**

The decline observed when only health care professionals accessed the data was no longer present once patients could monitor their own physical activity. Although seasonal influences cannot be excluded, these findings suggest that patient self-monitoring may support the maintenance of physical activity during hospital stays. Sustainability is complex, and determining the effect of patient self-monitoring alone remains challenging. Larger studies are needed to confirm these results.

## Introduction

Growing attention has been given to physical activity during hospital stays [1-4]. Inactivity is highly prevalent during hospitalization and is recognized by health care professionals as an important issue to be addressed [5-8]. Various interventions have been implemented in hospital settings to reduce this unwanted sedentary behavior, including strategies such as early mobilization and patient education [9-16].

Among these interventions, self-monitoring of physical activity is an effective strategy for improving patient activity during hospital stays [16-19]. By using movement monitors or accelerometers, clinicians can record patients’ activity levels throughout admission. These data are valuable both for clinicians, as an outcome measure, and for patients, as feedback on their progress and recovery [18,20-22]. In a recent pilot study, hospitalized patients undergoing cardiac rehabilitation were randomly assigned either to standard activity monitoring (with an accelerometer) or to an intervention combining activity monitoring with a physical activity dashboard and daily feedback [23]. The intervention group demonstrated significantly higher activity levels at discharge compared to the control group, showing the potential of physical activity monitoring during hospital stay.

However, once implemented, most interventions are rarely assessed to determine whether their effects are sustained over time [11,23-26]. Research in physical activity implementation has largely focused on effectiveness at specific time points using cross-sectional designs, providing limited insight into long-term sustainability [15,16]. However, sustainability is complex, and even perfectly delivered interventions may not thrive over time due to voltage drop (ie, the effectiveness diminishes in real-world conditions) or program drift (ie, the delivery gradually deviates from the intended protocol) [27]. Therefore, it is important to examine whether interventions designed to modify physical activity are maintained after their implementation. Implementation frameworks such as RE-AIM (Reach, Effectiveness, Adoption, Implementation, and Maintenance) are available to support such assessments, as they address not only how interventions are delivered but also the extent to which their use and effects are maintained over time [28]. In addition, analytical approaches such as interrupted time series (ITS) designs are particularly valuable in this context [29-31] as they allow for the assessment of both the immediate effects of an intervention and changes in trends over time.

Hence, this study investigated whether providing patients with real-time physical activity feedback, compared to receiving no real-time physical activity feedback, supported patients in maintaining physical activity levels in the cardiology ward. The evaluation was guided by the RE-AIM framework, with maintenance of the intervention as the primary outcome.

## Methods

### Study Design

This study used an ITS design using prospectively collected routine care data. A Hybrid Type 2 implementation-effectiveness approach was applied [32]. This allowed simultaneous evaluation of the self-monitoring intervention on daily physical activity and the implementation outcomes using the RE-AIM framework [32]. This study was reported in accordance with the SQUIRE (Standards for Quality Improvement Reporting Excellence) 2.0 [33] and STROBE (Strengthening the Reporting of Observational Studies in Epidemiology) guidelines [34].

### Intervention

The intervention consisted of the addition of a ward-based screen that provided patients with real-time feedback on their daily physical activity, introduced after a baseline phase as part of a 2-phase design.

In phase 1 (April 2024 to September 2024). During this first phase, only the health care practitioners (HCPs) of the cardiology ward had access to the patient’s daily physical activity data in the electronic health record (EHR).

In phase 2 (September 2024 to March 2025), a ward-based screen was added to supplement phase 1 activities, providing patients and HCPs with real-time insight into daily physical activity levels. Patients could view both their own daily physical activities and that of others in the ward. All patients were represented on the screen by an alias, ensuring that no real names were ever displayed. Patients could choose their own alias; if no preference was expressed, an alias was automatically assigned by the Atris system (Dawn Technology). The screen was positioned outside the patient rooms, directly in front of the ward’s secretary desk. The location and setting of the screen are shown in Figure 1. Details on ward type and typical patient admissions, provided together with the implementation strategies used, are shown in Multimedia Appendix 1.

It is important to note that phase 1 did not represent a routine care baseline. During phase 1, HCPs already had access to patients’ daily physical activity data via the EHR web viewer, and implementation strategies, including training of coaches and key users, and clinical lessons on the ward were already in place. The introduction of the ward-based screen in phase 2 was therefore the primary difference between phases. The comparison between phases reflected the effect of adding the ward screen to an already active implementation context, rather than a comparison between an intervention and a control period.

**Figure 1.**
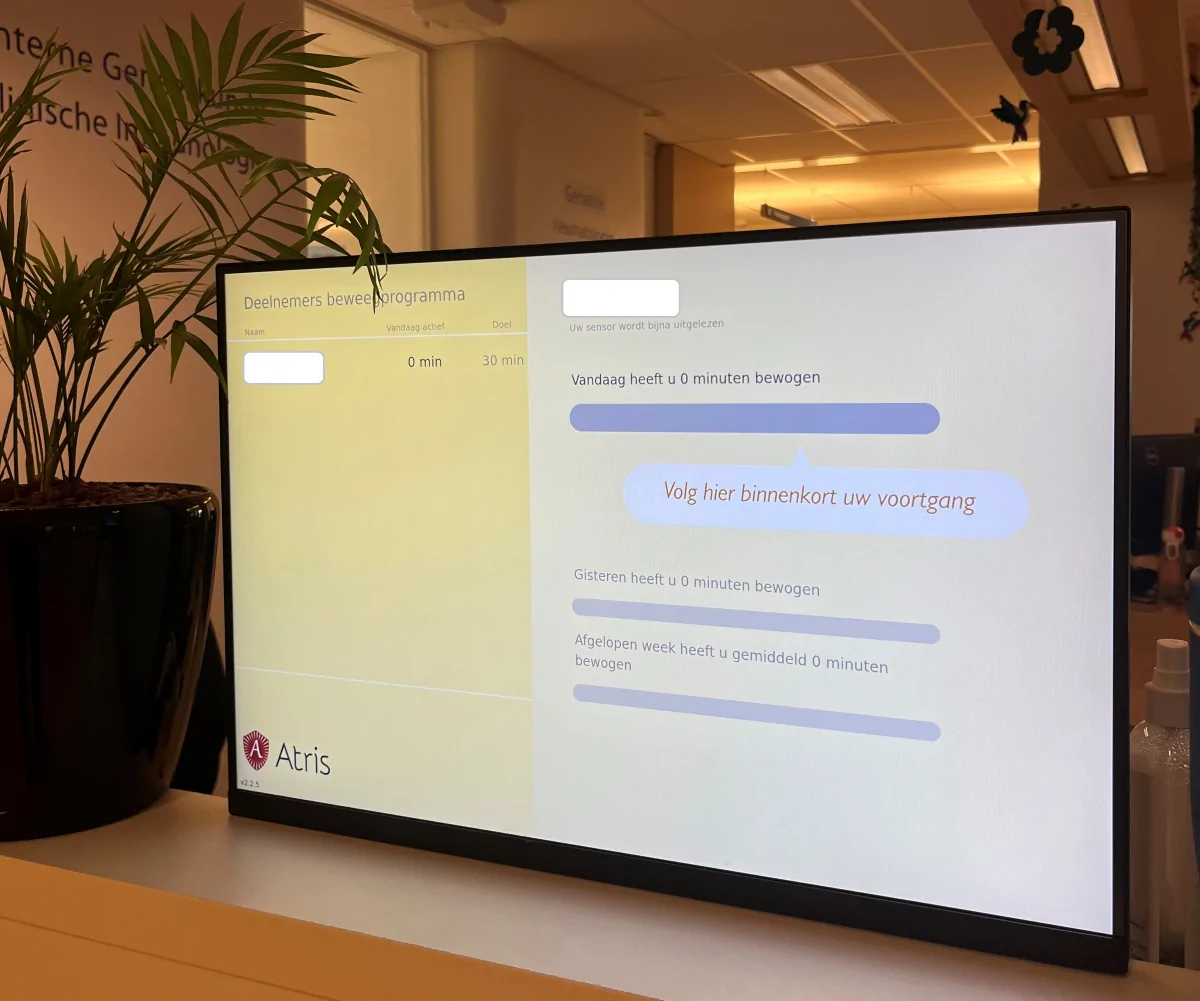
Example of the ward-based screen used for the self-monitoring intervention in the cardiology ward at the University Medical Center Utrecht (UMC Utrecht), the Netherlands, during phase 2 (September 2024 to March 2025). The screen, powered by the Atris platform, was positioned at the ward’s secretary desk. The left panel displays a group overview of patients identified by an alias alongside their total daily physical activity in minutes. The right panel shows an individual patient view with today’s, yesterday’s, and weekly average activity. No patient names were displayed. The “Doel” (Goal) column was not activated during the study period.

### Setting and Study Population

The self-monitoring intervention was implemented as part of routine care in the cardiology ward from the University Medical Centre of Utrecht (UMC), the Netherlands. Data were collected from patients and HCPs from the cardiology ward. Both patients and HCP had different data recorded and different inclusion and exclusion criteria. At the cardiology ward, patients with an expected hospital stay of at least 48 hours receive an accelerometer as part of routine care unless they are wheelchair-dependent. Patients were eligible for inclusion in the study when they were admitted to the cardiology ward and had at least 2 consecutive valid days of physical activity data recorded by the accelerometer. A valid day was considered any day that had at least 1 minute of total physical activity. The 1-minute threshold for a valid day was established based on consultation with HCPs and researchers with experience in the use of the Physical Activity Monitor (PAM) accelerometer in cardiology care. In regular clinical practice, patients recording 0 minutes of activity were found not to have worn the device at all, while any recording of at least 1 minute reliably indicated actual wear. This threshold therefore reflected a meaningful distinction between nonwear and wear days in this specific device and population context, rather than an arbitrary cutoff. Partial wear days, including admission and discharge days, were considered invalid measurement days, as variation in admission and discharge times could result in incomplete activity recordings that were not comparable across patients. Moreover, the requirement of at least 2 consecutive valid days was applied to ensure that the physical activity data represented a stable and meaningful period of the patient’s hospital stay, rather than isolated or incomplete observations. Patients were excluded if they had objected against the use of their routine care data for research purposes or had a hospital stay shorter than 48 hours. HCPs were included if they were a doctor, nurse, or physiotherapist working in the cardiology ward, they used or were currently using the accelerometers in cardiology care, and they had direct clinician-patient interactions.

### Ethical Considerations

The PAM accelerometers were implemented as part of routine care in the cardiology ward at the UMC Utrecht. Hence, this study did not fall under the scope of the Dutch Medical Research Involving Human Subjects Act (WMO) and therefore did not require approval from an accredited medical ethics committee. However, an independent quality check was conducted at UMC Utrecht to ensure compliance with applicable legislation and regulations, including informed consent procedures, data management, privacy, and legal requirements. Non-WMO permission was granted following protocol review (24U-0064-CARMMI). Hence, informed consent for the use of routine care data was waived. All patient data, including EHR data and accelerometer activity data, were pseudonymized prior to analysis, and no traceable information was available to the research team at any point. HCPs provided verbal consent for the collection of net promoter scores (NPSs) under the CARMMI study framework. Their data were also pseudonymized, with no traceable information available. No compensation was provided to patients or HCPs.

### Study Outcomes

Implementation outcomes were evaluated using the RE-AIM framework, which assesses Reach, Effectiveness, Adoption, Implementation, and Maintenance of the self-monitoring intervention [28]. Data for each RE-AIM dimension were collected across both phases of the implementation process (Table 1).

**Table 1.** Overview of the RE-AIM (Reach, Effectiveness, Adoption, Implementation, and Maintenance) framework components and corresponding outcome measures^a^.

RE-AIM model component	Outcome
Reach	Percentage of eligible patients that received an accelerometer
Effectiveness	Average daily physical activity of the patients during the implementation phases
Adoption	Net promoter score results
Implementation	Percentage of wearing time per patient
Maintenance	Trends of daily physical activity within each of the implementation phases

a These were used to evaluate a ward-based self-monitoring intervention for physical activity in inpatients receiving cardiology care at the University Medical Centre Utrecht (UMC Utrecht). Outcomes were assessed across 5 RE-AIM dimensions: Reach, Effectiveness, Adoption, Implementation, and Maintenance.

Reach was defined as the percentage of eligible patients admitted to the ward who were given an accelerometer. This was expressed as the percentage of patients who received the accelerometer divided by the total number of patients who were admitted to the ward during that phase. Eligible patients were those with a length of stay exceeding 48 hours who did not use wheelchairs. We prespecified ≥70% as the implementation target [16].

Effectiveness, defined as the impact of the intervention, was assessed by comparing average daily physical activity levels between phases, summarized with geometric means (GMs) due to skewed data. Physical activity data were measured with the PAM version AM400. The PAM is a 3-axial accelerometer, which is worn around the ankle and measures the amount of total activity time. Agreement with the well-established accelerometer Actigraph (wGT3X-Bt) showed a strong intraclass correlation coefficient of 0.955 in patients from different wards [35]. The PAM registers movements above the threshold of 1.4 metabolic equivalent task (MET), which are labeled as active minutes. The PAM provides a daily value of physical activity expressed in active minutes per day (eg, 17 minutes/day). This output was expressed as daily physical activity levels. HCPs did not perform any physical activity data entry or collection. Upon patient admission, ward staff connected the PAM, after which data collection began automatically. No further action was required from HCPs or patients beyond ensuring continued device wear. In addition, the following demographic and clinical characteristics of the patients were obtained to be used as covariates for the analysis: age (years), sex (male/female), height (cm), weight (kg), and BMI (kg/m^2^). Length of stay, discharge destination, and reason of admission were also retrieved from the EHR. To classify the reason for admission, admission diagnoses were categorized into clinical subgroups (eg, acute coronary syndromes, heart failure, cardiac rhythm disorders, and pericardial infection/inflammation) in collaboration with HCPs from the cardiology ward.

Adoption was defined within the RE-AIM framework as the willingness of HCPs to deliver the intervention. Therefore, HCPs were invited to complete a brief survey incorporating the NPS methodology [36]. The NPS survey is simple and widely used, but research in health care suggests its validity is modest and caution is needed when interpreting single-item scores [36,37]. HCPs were approached in person, and verbal consent was obtained prior to participation. The NPS survey was administered once during phase 1 to establish baseline perceptions and twice during phase 2 to monitor changes in HCPs’ experience over time. As the surveys were anonymous and completed by different HCPs on each occasion, these represented 3 independent cross-sectional measurements rather than a repeated-measures design. The survey consisted of 2 questions rated from 0 to 10: “On what level do you recommend the PAM to other colleagues?” and “On what level do you recommend the PAM to patients?” The surveys were completely anonymous, and different HCPs answered these surveys each time. Although the NPS is traditionally categorized into promoters (9-10), passives (7-8), and detractors (0‐6), with the final score calculated as the percentage of promoters minus detractors, this approach was not used in the present study. Instead, NPS responses were analyzed as a continuous variable on the original 0 to 10 scale, an approach commonly adopted for smaller samples [36]. Scores were summarized using means and SDs.

Implementation was defined as the extent to which the intervention was delivered as intended, that is, whether patients wore the accelerometer every day during their hospital admission. Therefore, implementation was expressed as the wearing time (percentage of wearing days per patient) in each of the phases. Because no consensus threshold exists for accelerometer adherence in hospitalized populations, we arbitrarily considered ≥60% of admission days as satisfactory. Therefore, the proportion of patients meeting this threshold in each phase was also reported.

Maintenance of the intervention, the primary outcome of the study, was evaluated as the trend in daily physical activity levels in the ward within each phase. This outcome therefore reflected the sustainability of the intervention by assessing whether average ward-level activity was maintained across phases and whether there were differences between them.

### Data Analyses

All analyses were conducted in Python (Python Software Foundation; version 3.11.9) using stats models, pandas, and SciPy. Days without accelerometer wear time were excluded from the analysis. For patients with admissions in both phase 1 and phase 2, only data from their first admission were included to minimize bias from prior exposure.

Baseline demographic and clinical characteristics were summarized separately for patients in phase 1 and phase 2. Continuous variables (ie, age, height, weight, BMI, length of stay, and wearing time) were reported as means and SDs. Group differences were tested using the Mann-Whitney *U* test. Categorical variables (ie, sex, discharge destination, and reason for admission) were reported as counts and percentages and compared across phases using chi-squared test.

Our primary outcome for the analysis was the RE-AIM component Maintenance. Because physical activity was log-normally distributed, we log-transformed values and fit an ITS using a linear mixed effects model with restricted maximum likelihood estimation to estimate within-phase trends and the immediate change at phase 2 onset (immediate change in physical activity at the beginning of phase 2) [31,38,39]. Fixed effects included time (days since the start of observation), phase (phase 1 vs phase 2), and their interaction. A random intercept was added for each patient to account for repeated measures. In this specification, the time coefficient represents the trend in phase 1. The phase coefficient captures the immediate level change at the start of phase 2, and the interaction term quantifies any change in trend following the intervention.

The secondary outcomes (the remaining RE-AIM components) were compared between the 2 phases. First, differences in Reach between phases were compared using a 2-proportion z-test. Second, to evaluate the Effectiveness outcome, we calculated the average treatment effect of the intervention on physical activity with a weighted least squares regression model. This model included inverse probability of treatment weighting (IPW) with propensity scores to adjust for baseline confounding. Propensity scores were calculated via logistic regression using baseline covariates. Weights were calculated as the inverse of the estimated probability of receiving the actual treatment. Standardized mean differences were calculated for all covariates before and after weighting to evaluate covariate balance. Physical activity, the dependent variable, was log-transformed, so model coefficients could be interpreted as geometric mean ratios.

Third, differences in Adoption, measured by NPS across phase 1, phase 2 (beginning), and phase 2 (end), were compared using a 1-way ANOVA, as normality assumptions were met. Fourth and last, Implementation between phases was evaluated using the Mann-Whitney *U* test due to nonnormal distribution. The proportion of patients meeting the threshold was compared using the chi-square test. A significance level of α=.05 was used for all statistical tests.

### Reflexivity

As researchers focused on physical activity during hospitalization, we acknowledge that we anticipated the addition of real-time physical activity self-monitoring would help maintain, and possibly increase, patient activity levels. To reduce the influence of these assumptions, all analyses were conducted using predefined procedures and independently reviewed by a statistician to ensure accurate interpretation. Although some authors (CR, KK, and GTJS) were involved in implementing the 2-phase intervention within the ward, the authors who led the design, interpretation, and writing of this paper (CdML, KV, and TJH) were not involved in daily cardiology ward procedures and did not influence clinical practice during the study period. Findings were discussed with the multidisciplinary team directly involved in implementation to ensure that multiple perspectives were considered.

## Results

### Baseline Characteristics

In total, data were recorded for 233 hospital admissions across the 2 phases. Of these, 9 patients had admissions in both phases, of whom 5 had three admissions across the study period, accounting for 14 duplicate admissions in total. Only the first admission per patient was retained, resulting in 219 unique patient admissions. Of these, 60 patients were excluded due to having fewer than 2 days of recorded daily physical activity, leading to a final sample of 159 patients included in the study. No further information was available regarding the reasons for discontinuous activity recordings or the absence of valid measurement days. In total, 75 patients had their daily physical activity recorded during phase 1 and 84 patients during phase 2 (Table 2). No significant differences were found regarding age (*P*=.15) and length of stay (*P*=.73) between patients from both phases. Patients in phase 2 had significantly lower BMI compared to patients in phase 1 (*P*=.01).

**Table 2.** Baseline characteristics of cardiology inpatients at the University Medical Centre Utrecht (UMC Utrecht) across 2 implementation phases: phase 1 (April 2024 to September 2024, n=75) and phase 2 (September 2024 to March 2025, n=84)^a^.

Outcome (statistic)	Phase 1 (n=75)	Phase 2 (n=84)	*P* value
Age, median (IQR)	66 (21‐88)	59 (18‐89)	.15
Weight, median (IQR)	82.5 (42.9‐169)	80 (44.3‐135)	.17
Height, median (IQR)	176 (140‐200)	177.5 (155-207)	.24
BMI, median (IQR)	26.9 (20.1‐43.6)	24.3 (16‐33.7)	.01^b^
Length of stay, median (IQR)	12 (2‐75)	12 (4-62)	.73
Sex, n (%)			.64
Female	24 (32)	23 (27.4)	
Male	51 (68)	61 (72.6)	
Reason for admission, n (%)			.75
Observations and general admission	8 (10.7)	9 (10.7)	
Cardiac arrest/resuscitation	5 (6.7)	10 (11.9)	
Pericardium infection/inflammation	5 (6.7)	5 (6)	
Myocardial ischemia and infarction	24 (32)	18 (21)	
Heart failure and myocardial insufficiency	21 (28)	25 (29.8)	
Valve abnormalities	2 (2.7)	2 (2.4)	
Rhythm and conduction disorders	10 (13.2)	15 (17.9)	
Discharge destination, n (%)			.64
Home	60 (80)	73 (86.9)	
Other hospital	6 (8)	6 (7.1)	
Nursing home	1 (1.3)	0 (0)	
Rehabilitation hospital	6 (8)	4 (4.8)	
Died in hospital	2 (2.7)	1 (1.2)	

a Values are presented as median (IQR) for continuous variables and n (%) for categorical variables. Between-phase differences were assessed using the Mann-Whitney *U* test for continuous variables and the chi-square test or Fisher exact test for categorical variables.

b *P*<.05 was considered statistically significant.

### Primary Outcome: Maintenance

The trend analysis of the restricted maximum likelihood model showed that daily physical activity levels of patients significantly declined during phase 1 (β=–0.002, *P*<.001), corresponding to an estimated 5.9% decrease of daily physical activity per month (exp (–0.002×365.25/12) ≈ 0.941).

During phase 2, the trend analysis showed that daily physical activity stabilized, and the trend was not significantly different from 0 (β=0.000). The time trends significantly differed between phases, with a positive interaction term (β=0.002, *P*=.03), indicating a flattening of the downward trend of daily physical activity in phase 2 (Table 3). The results of the physical activity trends per phase are shown in Figure 2. No statistically significant immediate change in daily physical activity levels was observed at the start of phase 2 right after the end of phase 1 (β=–0.127, *P*=.81). In addition, to account for the significant difference in baseline BMI between phases (*P*=.01), the primary model was additionally adjusted for BMI. The interaction term remained statistically significant (β=0.002, *P*=.02).

**Table 3.** Results of the linear mixed effects interrupted time series model for daily physical activity (log-transformed and non-transformed) across implementation phases among inpatients in cardiology care at the University Medical Centre Utrecht (UMC Utrecht) (phase 1: April 2024 to September 2024, n=75; phase 2: September 2024 to March 2025, n=84)^a^.

Fixed effects	Log-transformed total activity β^b^ (SE)	*z* ^c^	*P* value	Total activity β (SE)	*z*	*P* value	Log-transformed (BMI-adjusted^d^) β (SE)	*z*	*P* value
Intercept	3.710 (0.185)	20.025	<.001	54.442 (6.372)	8.544	<.001	4.309 (0.310)	13.919	<.001
Time index	–0.002 (0.001)	–3.720	<.001	–0.050 (0.019)	–2.572	.01	–0.002 (0.001)	–3.787	<.001
Phase 2	–0.127 (0.532)	–0.239	.81	–26.547 (18.570)	–1.430	.15	–0.250 (0.529)	–0.472	.64
Time × Phase 2	0.002 (0.001)	2.212	.03	0.071 (0.028)	2.545	.01	0.002 (0.001)	2.381	.02
BMI	—^e^	—	—	—	—	—	–0.022 (0.009)	–2.430	.02

a The time index reflects the within-phase daily trend, phase 2 reflects the level change at the introduction of the ward-based screen, and the Time × Phase 2 interaction reflects the difference in slope between phases.

b β: regression coefficient.

c *z*: *z* statistic.

d The BMI-adjusted model includes BMI as a covariate.

e Not applicable.

**Figure 2.**
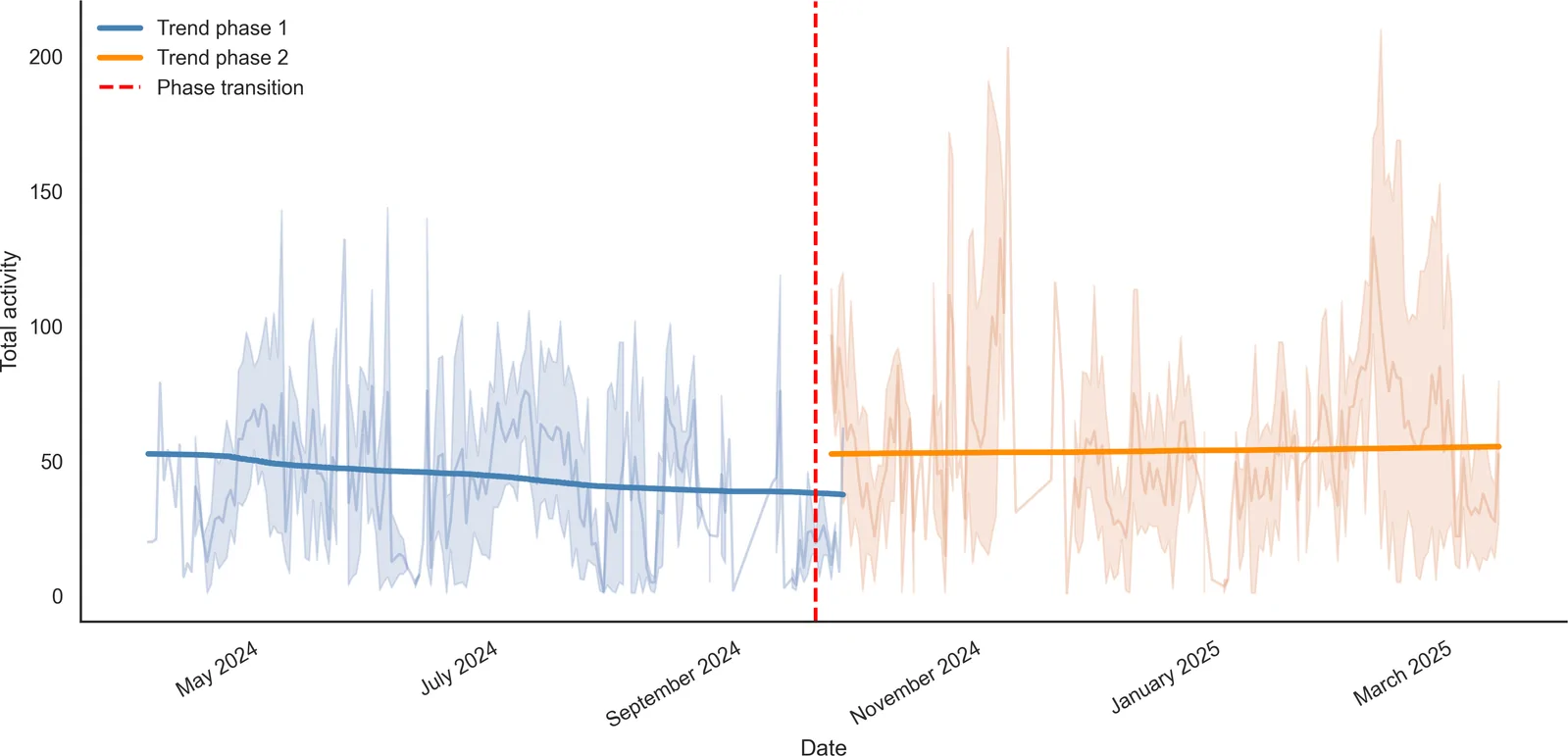
Trends in daily physical activity (min) across the 2 implementation phases of a ward-based physical activity self-monitoring intervention in cardiology inpatients at the University Medical Center Utrecht (UMC Utrecht), the Netherlands. Phase 1 (April 2024 to September 2024, n=75): health care practitioners had access to patient activity data via the electronic health record only. Phase 2 (September 2024 to March 2025, n=84): a ward-based screen additionally provided patients with real-time insight into daily physical activity. The y-axis displays individual patient-day observations; shaded areas represent the full distribution of observed values. Solid lines represent separate linear trends fitted for each phase. The vertical dashed red line marks the transition from phase 1 to phase 2. A declining trend was observed during phase 1 (β=–0.002, *P*<.001), followed by stabilization in phase 2 (β=0.000, *P*>.05).

Three sensitivity analyses were performed to assess the robustness of the primary outcome. First, to assess the potential influence of seasonality, a sensitivity analysis was conducted incorporating calendar month as a covariate in the primary model. Calendar month was not significantly associated with daily physical activity levels (β=0.001, *P*=.98), and the interaction term remained in the same direction (β=0.002). Second, to assess the influence of extreme activity values on the primary outcome, a sensitivity analysis was conducted excluding individual measurements exceeding 200 activity units (n=4 observations, 0.4% of the data, from 2 patients). The results were consistent with the primary analysis: daily physical activity significantly declined during phase 1 (β=–0.002, *P*<.001), stabilized during phase 2 (net slope≈0.000), and the interaction term remained statistically significant (β=0.002, *P*=.02). Third and last, to assess the potential influence of patients with extended length of stay on the observed activity trajectories, a sensitivity analysis was conducted excluding patients with a length of stay exceeding 30 days (n=14 patients, 193 observations). The results remained consistent with the primary analysis: daily physical activity significantly declined during phase 1 (β=–0.002, *P*<.001), stabilized during phase 2 (net slope≈0.000), and the interaction term remained statistically significant (β=0.002, *P*=.04).

The results of the ITS primary analysis are presented in Table 3. The results based on nontransformed daily physical activity were consistent with the log-transformed model, supporting the robustness of the findings. Observed total activity measurements and corresponding fitted values over time are shown in Multimedia Appendix 2.

### Secondary Outcomes

For Reach, in phase 1, 36.6% (105/287 who were eligible based on length of stay) of admitted patients received an accelerometer, compared to 36.2% (128/354) in phase 2. This difference was not statistically significant (*P*=.91).

For Effectiveness, the GM of daily physical activity was 20.5 minutes for phase 1 and 23.5 minutes for phase 2. The exponentiated coefficient of the treatment effect (β=0.132) yielded a geometric mean ratio of 1.141, indicating a 14.1% higher average daily physical activity among patients in phase 2 compared to those in phase 1. However, this difference was not statistically significant (*P*=.52, 95% CI 0.761‐1.709). The results of the IPW are shown in Multimedia Appendix 3.

For Adoption, mean recommendation scores to colleagues were 7.1 (SD 1.30) in phase 1 (n=17), 7.2 (SD 1.40) in the beginning of phase 2 (n=10), and 6.8 (SD 1.22) at the end of phase 2 (n=19). One-way ANOVA indicated no statistically significant differences in recommendation to colleagues between phases (*F*_2,43_=0.332, *P*=.72). The mean recommendation scores to patients were 7.4 (SD 1.3) in phase 1, 7.5 (SD 1.7) in the beginning of phase 2, and 6.5 (SD 1.1) at the end of phase 2. These differences were also not statistically significant (*F*_2,43_=2.900, *P*=.07).

Fourth and final, for Implementation, the median wearing time in phase 1 was 60.0% (IQR 42.0%‐74.5%) compared to 66.7% (IQR 50.0%‐78.9%) in phase 2; this difference was not statistically significant (Mann-Whitney *U*=3563.5, *P*=.15). Regarding the predefined implementation threshold of ≥60% wearing time, 52 of 75 (69.3%) patients in phase 1 and 52 of 84 (61.9%) patients in phase 2 met this threshold, with no statistically significant difference between phases (*χ*²_1_=0.666, *P*=.41). The results of the secondary analyses are displayed in Table 4.

**Table 4.** Summary of implementation outcomes evaluated using the RE-AIM (Reach, Effectiveness, Adoption, and Implementation) framework across 2 phases of a ward-based physical activity self-monitoring intervention in cardiology inpatients at the University Medical Centre Utrecht (UMC Utrecht), the Netherlands (phase 1: April 2024 to September 2024, n=75; phase 2: September 2024 to March 2025, n=84)^a^.

RE-AIM^b^ dimension and outcome	Phase 1	Phase 2	*P* value
Reach			
Eligible patients, %	36.6	36.2	.91
Effectiveness			
Changes in average daily physical activity levels (min)	20.5	23.5	.52
Adoption, mean (SD)			
NPS^c^ colleagues	7.1 (1.3)	7.2 (1.4)‐6.8 (1.2)^d^	.72
NPS patients	7.4 (1.3)	7.5 (1.7)‐6.5 (1.1)^d^	.07
Implementation, median (IQR)			
Percentage of wearing time	60.0 (42.0‐74.5)	66.7 (50.0‐78.9)	.15
Maintenance			
Trends of daily physical activity			.03^e^
Trend coefficient (β)	–0.002	0.000	
Activity change per month, %	–5.9	0.0	

a Reach was defined as the percentage of eligible patients who received an accelerometer. Effectiveness reflects the geometric mean of daily physical activity in minutes, derived from a weighted least squares model on log-transformed values. Adoption was measured using the net promoter score (NPS) methodology, reported as mean (SD) for recommendations to colleagues and patients separately, and compared across 3 time points using one-way ANOVA. Implementation reflects the percentage of wearing time per patient, reported as median (IQR) and compared using the Mann-Whitney *U* test. Maintenance reflects trends in daily physical activity.

b RE-AIM: Reach, Effectiveness, Adoption, Implementation, and Maintenance.

c NPS: net promoter score.

d Phase 2 values: beginning (n=10) and end (n=19).

e *P*<.05 is significant.

## Discussion

### Sumary of Outline

A ward-based screen that provided patients with real-time physical activity feedback was introduced after a baseline phase as part of a 2-phase design. In both phases, patients at the cardiology ward wore an accelerometer. The key distinction in phase 2 was the addition of visual feedback via a ward-based screen, which allowed patients to visualize both their own daily physical activity and that of other patients.

### Principal Findings

The results of the primary outcome, Maintenance, showed that daily physical activity levels of the ward declined statistically significantly over time in phase 1. During this first phase, patients did not have access to their daily physical activity data unless an HCP explicitly shared it with them. However, accessing this information required HCPs to navigate to a specific setting in the EHR, which was likely not done consistently. This suggests that simply providing HCPs with access to patients’ daily physical activity data via the EHR was possibly insufficient to sustain physical activity in the ward. In contrast, in phase 2, the negative time trend of daily physical activity was no longer observed. The addition of the ward-based screen where patients could visualize their physical activity was associated with a stabilization of physical activity levels. The addition of the ward-based screen possibly led to maintained levels of daily physical activity throughout phase 2, which lasted almost 6 months (24 wk). A possible explanation for this finding is that the introduction of the screen in the ward improved patients’ intrinsic motivation. Providing patients access to their daily activity levels supports self-monitoring, a known behavior-change technique that increases awareness of mobility [10,15,40,41].

Regarding the other RE-AIM framework outcomes, no statistically significant differences were found. First, in terms of Reach, only slightly more than one-third of patients with an expected hospital stay of at least 2 days received an accelerometer. This proportion did not meet the predefined implementation threshold and is considered a major limitation in the implementation of an intervention [42]. The reasons for the low Reach remain unclear, as the research team was not involved in the process of providing accelerometers to patients or coordinating their allocation. It is possible that accelerometer distribution was influenced by factors such as nursing workload, shift variability, or patients’ clinical status at the time of admission, which may have introduced selection bias. For example, patients who were less mobile, more acutely ill, or had a greater comorbidity burden may have been less likely to receive an accelerometer. Hence, the analyzed cohort may overrepresent patients with better functional status, potentially leading to an overestimation of ward-level physical activity.

Second, under the Effectiveness outcome, the 14.1% increase in daily physical activity from phase 1 to phase 2 was not statistically significant and may be due to chance. Still, the change could be clinically relevant. As previously mentioned, in hospitalized patients, even modest increases in daily physical activity have been associated with improved functional outcomes, shorter lengths of stay, and reduced risk of readmission [43,44]. For instance, Gallardo-Gómez et al [45] conducted a systematic review and meta-analysis evaluating the effects of various daily physical activity interventions on functional outcomes during hospitalization. They identified a minimal effective dose of daily physical activity during hospitalization at approximately 100 METs-minutes/day, which corresponds to 40 minutes of light activity or 25 minutes of moderate activity per day. In our study, although direct comparisons are limited due to differences in measurement methods (accelerometer vs MET-based estimation), the GM of physical activity during phase 2 reached approximately 23.5 minutes per day. This level closely approaches the 25-minute/day threshold identified in the literature as the minimal effective dose to reduce functional decline. Therefore, it is possible that a portion of patients in phase 2 reached or exceeded this threshold, and that the observed increase in daily physical activity may have contributed to a reduced risk of functional deterioration in these patients. In addition, it is important to note that this study did not include a power calculation, as the sample size was determined by the available data from the implementation period. Hence, the study may have been underpowered to detect small but clinically meaningful differences in daily physical activity between phases, and the nonsignificant effectiveness finding should be interpreted with this limitation in mind.

Third, regarding Adoption, NPS scores were similar across phases (around 7/10), indicating no improvement in HCP engagement. Scores of this level are considered modest and suggest limited support or promotion of the intervention by HCPs. While NPS is a quick and accessible tool for assessing stakeholder (HCPs) support, its simplicity may limit its ability to capture more subtle aspects of HCP engagement [36]. It is possible that changes in engagement occurred but were not detected due to the tool’s limitations. More detailed or sensitive evaluation methods may have offered deeper insight into HCP adoption. On the other hand, as HCPs also had direct insight into the patients’ daily physical activity after the addition of the screen, this could have enabled HCPs to encourage physical activity more actively, thereby increasing patients’ extrinsic motivation. Additionally, the visibility of other patients being active may have started a positive behavioral cascade within the cardiology ward [42,46,47]. As patients observed others engaging in physical activity, they may have felt inspired to do the same, reinforcing a culture of mobility and activity.

HCP engagement was considered closely related to the fourth and final RE-AIM outcome, Implementation. Implementation was measured as adherence. In phase 1, the mean wearing time was slightly below the predefined implementation threshold, whereas in phase 2 the mean exceeded the 60% threshold, indicating some improvement. We hypothesized that the screen would improve adherence by serving as a visual reminder, leading to HCPs to encourage patients to wear the device and increasing awareness when patients were not wearing it. However, results for wearing time remained stable across phases, with slightly higher percentages of wearing days in phase 2 compared to phase 1. While accelerometer-based self-monitoring has been shown to increase adherence outside the hospital setting [6,48], this effect was not clearly observed in our study. This may be related to the operational structure of the ward, particularly regarding accelerometer (PAM) ownership and responsibility. In the cardiology ward, patients with short hospital stays (less than 48 h) did not receive an accelerometer, meaning not all patients on the ward wore one. It is possible that HCPs did not consistently notice when data were missing or did not feel responsible for ensuring that patients were wearing the device.

### Comparison to Previous Work

It is important to recognize that the goal of feedback-based monitoring is not only to increase daily physical activity, but also to promote independent mobility [19,48,49]. A systematic review from Compernolle et al [40] showed that the use of objective self-monitoring tools, such as accelerometers, may improve adherence and support progress toward behavior change goals through awareness of inactivity. However, changes in patient inactivity require intentional application of behavior change techniques (BCTs). Interventions using activity trackers during hospital care have shown greater effectiveness in increasing physical activity levels compared to routine care [41]. However, as shown by Leeuwerk et al [10], the effectiveness of such interventions is closely linked to the type and number of BCTs used. Evidence suggests that interventions grounded in behavioral theory, which incorporate multiple BCTs and are paired with professional coaching, are more likely to produce meaningful increases in daily physical activity levels. In our case, the intervention involved solely the addition of a ward-based screen to provide real-time physical activity self-monitoring. This may explain the limited changes observed in the trends of phase 2. It is also possible that the addition of the ward-based screen may also have engaged other BCTs not originally anticipated, including peer comparison, self-monitoring, and intrinsic goal setting.

To date, there are no studies that evaluate changes in daily physical activity during hospital stays using an ITS design. Most ITS studies have been conducted in community settings, aiming to improve physical activity and reduce sedentary behavior in specific communities or patient groups [29,30,50,51]. Although pre-post analysis is a commonly used statistical approach, it does not capture how effects of an intervention on physical activity are maintained over time. ITS analysis was more suitable in this context, particularly for a Hybrid Type 2 design, where the aim was to simultaneously assess both the effect of the intervention and its implementation. In this study, ITS allowed us to model average changes and trends in daily physical activity across both phases and to estimate changes attributable to the intervention.

### Strengths and Limitations

This study had several strengths. First, physical activity was objectively measured using accelerometers, reducing self-report bias. Second, the study was conducted in a real-world clinical environment, enhancing its generalizability. Third and last, multidisciplinary involvement in the design and interpretation of the study strengthened the fidelity of the implementation and the interpretation of the findings.

First, data from routine care often involve a high proportion of missing data. While this reflects the realities of clinical practice, it presents certain limitations. We chose to exclude nonwearing days from the analyses, whereas current research increasingly supports the use of imputation methods, particularly when the proportion of missing data is not substantial. In our study, 60 (27.4%) patients were excluded for not meeting the minimum wear criterion. This exclusion occurred prior to analysis and reflects an inclusion criterion rather than missing data within the included sample. Among the 159 patients included in the final analysis, completeness was high. As missingness was limited, we used complete-case analysis instead of multiple imputation, assuming data were missing at random [52]. We therefore reported analyses based on observed values, which reflect the real conditions during hospitalization. Second, it would have been valuable to conduct qualitative interviews to gain deeper insights into HCPs’ experiences during the implementation process. However, this was not within the scope of the study, which was designed to focus strictly on RE-AIM outcomes and the quantitative data available. As a result, our evaluation was limited to the data that was collected. Third, the study did not include a comparable ward as a control group. This would have allowed for the comparison of outcomes across different settings. It could also have provided insight into implementation among different patient populations. Fourth, a potential limitation of this study is that the observed outcomes could partly reflect seasonal or temporal factors. These could include unmeasured secular trends, such as changes in ward protocols or other unmeasured co-interventions occurring during the study period, rather than the intervention itself. Although IPW was used to account for measured baseline differences between phases, it does not fully address temporal confounding arising from the sequential nature of phase 1 and phase 2. The ITS analysis and the sensitivity analysis incorporating calendar month as a covariate partially mitigated this limitation by explicitly modeling the underlying activity trend over time, independently of phase assignment.

Finally, several potential confounders should be acknowledged. Cognitive function was not included as an exclusion criterion, and patients with cognitive impairment may have been less able to recognize and interpret their own activity data, potentially reducing the intervention effect. In addition, differences in disease severity may have influenced both baseline activity levels and the capacity to respond to the intervention. Disease severity may also have affected activity restrictions, such as prescribed bed rest or limitations on mobility. However, these factors were not systematically recorded in the EHR and therefore could not be accounted for in the analysis.

### Future Directions

Future studies could explore incorporating additional BCTs to strengthen the self-monitoring intervention and further support physical activity maintenance during hospitalization. Additionally, future research should consider including information on the functional status and physical activity levels of patients prior to admission, as these factors will likely determine their activity upon hospitalization. These studies should move beyond recording total physical activity levels to also capture inactivity patterns, including sedentary bouts and moments of inconsistent activity. The relative effects of prolonged sedentary behavior and different patterns of physical activity accumulation remain poorly understood in hospitalized patients. For example, it is unclear whether 20 consecutive minutes of activity provides similar benefits to the same amount of activity accumulated through shorter bouts distributed throughout the day. Finally, future studies should examine within-day patterns of physical activity during hospitalization to better understand how real-time feedback on activity and inactivity, rather than daily activity totals alone, influences patient behavior and physical activity levels.

### Conclusions

This study investigated whether providing patients with real-time physical activity feedback supported the maintenance of activity levels in the cardiology ward compared with receiving no feedback. Daily ward physical activity levels were maintained following the introduction of the ward-based screen, suggesting that real-time access to activity data may support physical activity maintenance during hospitalization.

## Supplementary material

10.2196/92189 Multimedia Appendix 1 Cardiology ward description and implementation strategies.

10.2196/92189 Multimedia Appendix 2 Observed total daily physical activity (min) and model-fitted values from the linear mixed effects interrupted time series model across 2 implementation phases of a ward-based physical activity self-monitoring intervention in cardiology inpatients at the University Medical Centre Utrecht (UMC Utrecht), the Netherlands (phase 1: April 2024-September 2024; phase 2: September 2024-March 2025).

10.2196/92189 Multimedia Appendix 3 Covariate balance before and after inverse probability weighting.

## Abbreviations

BCT: behavior change techniques
EHR: electronic health record
GM: geometric mean
HCP: health care practitioner
IPW: inverse probability weighting
ITS: interrupted time series
MET: metabolic equivalent task
NPS: net promoter score
PAM: Physical Activity Monitor (PAM AM400)
RE-AIM: Reach, Effectiveness, Adoption, Implementation, Maintenance
SQUIRE: Standards for Quality Improvement Reporting Excellence
STROBE: Strengthening the Reporting of Observational Studies in Epidemiology
UMC: University Medical Centre
WMO: Dutch Medical Research Involving Human Subjects Act
