# Supplementary material for: Real-Time Physical Activity Self-Monitoring in Patients Hospitalized for Cardiology Care: Interrupted Time Series Analysis
Source: JMIR Cardio. 2026 Oct 7;10:e92189. doi: 10.2196/92189 (PMC13644949; doi:10.2196/92189)
Supplement: Supplementary file 1 [file cardio-v10-e92189-s001.docx]

## **Appendix 1**

**Cardiology ward description and implementation strategies**

The study was conducted in the cardiology ward of a university hospital in Utrecht, The Netherlands, which specializes in the diagnosis and treatment of acute and chronic cardiovascular conditions. The ward has a capacity of 30 inpatient beds and is staffed by a multidisciplinary team of approximately 60 nurses, with around 15 nurses present during the day shift. Daytime medical care is provided by six cardiology residents (assistant cardiologists) and four cardiology specialists who are assigned daily ward duties. In addition, two to three physiotherapists are routinely involved in patient care. The ward admits approximately 50–60 patients per month with a hospital stay exceeding 48 hours. Admissions primarily involve patients with cardiovascular conditions such as acute coronary syndromes, heart failure exacerbations, and cardiac rhythm disorders. The patient population predominantly consists of older adults with a high burden of cardiovascular risk factors and comorbidities, requiring both medical treatment and support for lifestyle-related behaviors, including physical activity during hospitalization.

Implementation strategies for the self-monitoring intervention

The intervention was introduced in two phases.

**Phase 1- (April 2024-September 2024)**

- Patients admitted to the Cardiology ward with an expected length of stay longer than 48 hours were given an accelerometer.
- During this first phase, only the healthcare practitioners (HCPs) of the Cardiology ward had access to the patient’s daily physical activity data via a web viewer in the Electronic Health Record (EHR).

**Phase 2 (September 2024-March 2025)**

- The screen was added to the ward to supplement Phase 1 activities, providing patients and HCPs with real-time insight into daily physical data.
- Patients could view both their own daily physical activity and that of others in the ward.
- All patients were represented on the screen by an alias, ensuring that no real names were ever displayed. Patients could choose their own alias; if no preference was expressed, an alias was automatically assigned by the Atris system.
- The screen was positioned outside the patient’s room, directly in front of the ward's secretary desk.

The system was largely automated. On admission, cardiology-ward staff provided each patient with an accelerometer (PAM) to be worn for the entire hospital stay and connected it to the digital platform. The device then automatically synchronized with the Atris system to record physical activity; patients only needed to keep wearing it. To monitor and ensure reliable implementation, we appointed coaches and key users.

- Coaches had expertise with the movement-monitoring system and knew how to operate the accelerometers and connect it to the digital platform (Atris). Coaches included physiotherapists, administrative staff, and project leaders. They provided a manual to key users to help resolve questions, and, together with the project manager, delivered clinical lessons on the ward to HCPs about the monitoring system. After the intervention was implemented, coaches checked in regularly with key users to evaluate progress and support the process.
- Key users (nurses, doctors, physiotherapists, and secretaries) led the on-ward implementation after being trained by the coaches on the accelerometer and related systems (ICT, PAMs, etc.). They acted as ambassadors for implementation across specialties and were responsible for successful adoption. Once trained, key users shared information with colleagues by teaching the tasks they had learned from the coaches. They also explained the intervention and project goals in daily and weekly meetings and set reminders for other HCPs
