# Supplementary material for: Real-Time Physical Activity Self-Monitoring in Patients Hospitalized for Cardiology Care: Interrupted Time Series Analysis
Source: JMIR Cardio. 2026 Oct 7;10:e92189. doi: 10.2196/92189 (PMC13644949; doi:10.2196/92189)
Supplement: Supplementary file 2 [file cardio-v10-e92189-s003.docx]

## **Appendix 3**

**Table 1.** Covariate Balance Before and After Inverse Probability Weighting.

| **Covariate** | **SMD Before** | **SMD After** |
| --- | --- | --- |
| Age | –0.211 | 0.029 |
| Gender (Male) | 0.101 | –0.029 |
| BMI | –0.440 | –0.030 |
| Length of Stay | –0.094 | 0.027 |
| Discharged Home | 0.185 | –0.003 |
| Discharged to Hospital | –0.032 | –0.012 |
| Discharged to Rehabilitation Hospital | –0.178 | 0.013 |
| Heart Failure and Myocardial Insufficiency | 0.039 | 0.063 |
| Myocardial Ischemia and Infarction | –0.239 | –0.028 |
| Observations and General Admission | 0.002 | 0.039 |
| Pericardium Infection/Inflammation | –0.029 | –0.006 |
| Rhythm and conduction disorders | 0.124 | –0.029 |
| Valve abnormalities | –0.018 | –0.004 |

Covariate Balance Before and After Inverse Probability Weighting. Discharge destination categories with very small cell sizes, specifically Nursing Home (n=1 and n=0) and Died in Hospital (n=2 and n=1) in Phase 1 and Phase 2 respectively , were excluded from the propensity score model to avoid instability in the inverse probability weighting estimation. Cardiac Arrest/Resuscitation served as the reference category for the reason for admission variable and is therefore not displayed as a separate covariate.
